# Supplementary material for: Prevalence and sociodemographic determinants of suboptimal glycemic control in persons with diabetes in Ghana: A systematic review and meta-analysis
Source: PLoS One. 2025 Jul 18;20(7):e0327610. doi: 10.1371/journal.pone.0327610 (PMC12273950; doi:10.1371/journal.pone.0327610)
Supplement: S1 Text — (DOCX) [file pone.0327610.s002.docx]

PubMed = 298

| Concept | Seach string |
| --- | --- |
| #1 Glycemic control | "Glycemic Control"[Mesh] OR "Blood Glucose"[Mesh] OR "Glycaemic control" OR "Glycemic control" OR "Glycated hemoglobin" OR "Glycated haemoglobin" OR "Glycosylated hemoglobin" OR "Glycosylated haemoglobin" OR "Hemoglobin A1c" OR "Haemoglobin A1c" OR HbA1c OR A1c OR GHb OR "Blood glucose" OR "Blood glucose control" OR "Blood glucose levels" OR "Blood sugar levels" OR "Fasting blood glucose" OR "Fasting blood sugar" OR "Fasting plasma glucose" OR FBS OR FBG OR FPG |
| #2 Diabetes | "Diabetes Mellitus"[Mesh] OR "Diabetes Mellitus, Type 2"[Mesh] OR "Diabetes Mellitus, Type 1"[Mesh] OR "Diabetes mellitus" OR Diabetes OR "Diabetic*" OR "Type 2 diabetes mellitus" OR "Type 2 diabetes" OR T2DM OR T2D OR DM2 OR "Non-insulin-dependent diabetes mellitus" OR NIDD OR "Type 1 diabetes mellitus" OR "Type 1 diabetes" OR T1DM OR T1D OR DM1 OR "Insulin-dependent diabetes" OR IDD |
| #3 Ghana | "Ghana"[Mesh] OR Ghana OR Ghanaian* |
| **#4** | #1 AND #2 AND #3 |

Medline = 193

| Concept | Seach string |
| --- | --- |
| #1 Glycemic control | exp Glycemic Control/ or exp Blood Glucose/ or exp Glycated Hemoglobin/ or ("Glycaemic control" or "Glycemic control" or "Glycated hemoglobin" or "Glycated haemoglobin" or "Glycosylated hemoglobin" or "Glycosylated haemoglobin" or "Hemoglobin A1c" or "Haemoglobin A1c" or HbA1c or A1c or GHb or "Blood glucose" or "Blood glucose control" or "Blood glucose levels" or "Blood sugar levels" or "Fasting blood glucose" or "Fasting blood sugar" or "Fasting plasma glucose" or FBS or FBG or FPG).mp. |
| #2 Diabetes | exp Diabetes Mellitus/ or exp Diabetes Mellitus, Type 2/ or exp Diabetes Mellitus, Type 1/ or ("Diabetes mellitus" or Diabetes or "Diabetic*" or "Type 2 diabetes mellitus" or "Type 2 diabetes" or T2DM or T2D or DM2 or "Non-insulin-dependent diabetes mellitus" or NIDD or "Type 1 diabetes mellitus" or "Type 1 diabetes" or T1DM or T1D or DM1 or "Insulin-dependent diabetes" or IDD).mp. |
| #3 Ghana | exp Ghana/ or (Ghana or Ghanaian*).mp. |
| **#4** | #1 AND #2 AND #3 |

Embase = 325

| Concept | Seach string |
| --- | --- |
| #1 Glycemic control | exp glycemic control/ or exp glucose blood level/ or ("Glycaemic control" or "Glycemic control" or "Glycated hemoglobin" or "Glycated haemoglobin" or "Glycosylated hemoglobin" or "Glycosylated haemoglobin" or "Hemoglobin A1c" or "Haemoglobin A1c" or HbA1c or A1c or GHb or "Blood glucose" or "Blood glucose control" or "Blood glucose levels" or "Blood sugar levels" or "Fasting blood glucose" or "Fasting blood sugar" or "Fasting plasma glucose" or FBS or FBG or FPG).mp. |
| #2 Diabetes | exp diabetes mellitus/ or exp non insulin dependent diabetes mellitus/ or exp insulin dependent diabetes mellitus/ or ("Diabetes mellitus" or Diabetes or "Diabetic*" or "Type 2 diabetes mellitus" or "Type 2 diabetes" or T2DM or T2D or DM2 or "Non-insulin-dependent diabetes mellitus" or NIDD or "Type 1 diabetes mellitus" or "Type 1 diabetes" or T1DM or T1D or DM1 or "Insulin-dependent diabetes" or IDD).mp. |
| #3 Ghana | exp Ghana/ or (Ghana or Ghanaian*).mp. |
| **#4** | #1 AND #2 AND #3 |

Global Health = 162

| Concept | Seach string |
| --- | --- |
| #1 Glycemic control | (blood sugar or haemoglobin A1 or hyperglycaemia).sh. or ("Glycaemic control" or "Glycemic control" or "Glycated hemoglobin" or "Glycated haemoglobin" or "Glycosylated hemoglobin" or "Glycosylated haemoglobin" or "Hemoglobin A1c" or "Haemoglobin A1c" or HbA1c or A1c or GHb or "Blood glucose" or "Blood glucose control" or "Blood glucose levels" or "Blood sugar levels" or "Fasting blood glucose" or "Fasting blood sugar" or "Fasting plasma glucose" or FBS or FBG or FPG).mp. |
| #2 Diabetes | exp diabetes/ or exp diabetes mellitus/ or exp type 2 diabetes/ or exp type 1 diabetes/ or ("Diabetes mellitus" or Diabetes or "Diabetic*" or "Type 2 diabetes mellitus" or "Type 2 diabetes" or T2DM or T2D or DM2 or "Non-insulin-dependent diabetes mellitus" or NIDD or "Type 1 diabetes mellitus" or "Type 1 diabetes" or T1DM or T1D or DM1 or "Insulin-dependent diabetes" or IDD).mp. |
| #3 Ghana | exp Ghana/ or (Ghana or Ghanaian*).mp. |
| **#4** | #1 AND #2 AND #3 |

Scopus = 242

| Concept | Seach string |
| --- | --- |
| #1 Glycemic control | TITLE-ABS-KEY ("Glycaemic control" OR "Glycemic control" OR "Glycated hemoglobin" OR "Glycated haemoglobin" OR "Glycosylated hemoglobin" OR "Glycosylated haemoglobin" OR "Hemoglobin A1c" OR "Haemoglobin A1c" OR HbA1c OR A1c OR GHb OR "Blood glucose" OR "Blood glucose control" OR "Blood glucose levels" OR "Blood sugar levels" OR "Fasting blood glucose" OR "Fasting blood sugar" OR "Fasting plasma glucose" OR FBS OR FBG OR FPG) |
| #2 Diabetes | TITLE-ABS-KEY ("Diabetes mellitus" OR Diabetes OR "Diabetic*" OR "Type 2 diabetes mellitus" OR "Type 2 diabetes" OR T2DM OR T2D OR DM2 OR "Non-insulin-dependent diabetes mellitus" OR NIDD OR "Type 1 diabetes mellitus" OR "Type 1 diabetes" OR T1DM OR T1D OR DM1 OR "Insulin-dependent diabetes" OR IDD) |
| #3 Ghana | TITLE-ABS-KEY (Ghana OR Ghanaian*) |
| **#4** | #1 AND #2 AND #3 |

Web of science = 156

| Concept | Seach string |
| --- | --- |
| #1 Glycemic control | TOPIC ("Glycaemic control" OR "Glycemic control" OR "Glycated hemoglobin" OR "Glycated haemoglobin" OR "Glycosylated hemoglobin" OR "Glycosylated haemoglobin" OR "Hemoglobin A1c" OR "Haemoglobin A1c" OR HbA1c OR A1c OR GHb OR "Blood glucose" OR "Blood glucose control" OR "Blood glucose levels" OR "Blood sugar levels" OR "Fasting blood glucose" OR "Fasting blood sugar" OR "Fasting plasma glucose" OR FBS OR FBG OR FPG) |
| #2 Diabetes | TOPIC ("Diabetes mellitus" OR Diabetes OR "Diabetic*" OR "Type 2 diabetes mellitus" OR "Type 2 diabetes" OR T2DM OR T2D OR DM2 OR "Non-insulin-dependent diabetes mellitus" OR NIDD OR "Type 1 diabetes mellitus" OR "Type 1 diabetes" OR T1DM OR T1D OR DM1 OR "Insulin-dependent diabetes" OR IDD) |
| #3 Ghana | TOPIC (Ghana OR Ghanaian*) |
| **#4** | #1 AND #2 AND #3 |
