# Supplementary material for: Prevalence and sociodemographic determinants of suboptimal glycemic control in persons with diabetes in Ghana: A systematic review and meta-analysis
Source: PLoS One. 2025 Jul 18;20(7):e0327610. doi: 10.1371/journal.pone.0327610 (PMC12273950; doi:10.1371/journal.pone.0327610)
Supplement: S1 Table — (DOCX) [file pone.0327610.s003.docx]

Quality assessment of included studies using (JBI Checklist for Prevalence Studies)

| First author (year) | Was the sample frame appropriate to address the target population? | Were study participants sampled in an appropriate way? | Was the sample size adequate? | Were the study subjects and the setting described in detail? | Was the data analysis conducted with sufficient coverage of the identified sample? | Were valid methods used for the identification of the condition? | Was the condition measured in a standard, reliable way for all participants? | Was there appropriate statistical analysis? | Was the response rate adequate, and if not, was the low response rate managed appropriately? | Score |
| --- | --- | --- | --- | --- | --- | --- | --- | --- | --- | --- |
| Afaya (2020) | Yes | No | Yes | Yes | Yes | Yes | Unclear | Yes | Yes | 7 |
| Sefah (2020) | Yes | Yes | Yes | Yes | Yes | Yes | Unclear | Yes | Yes | 8 |
| Botchway (2022) | Yes | No | Unclear | Yes | Yes | Yes | Yes | Yes | Unclear | 6 |
| Mobula (2018) | Yes | No | Yes | Yes | No | Yes | Yes | Yes | Unclear | 6 |
| Alor (2023) | Yes | Yes | Yes | Yes | Yes | Yes | Unclear | Yes | Yes | 8 |
| Agyekum (2023) | Yes | Yes | Yes | Yes | Yes | Yes | Yes | Yes | Yes | 9 |
| Fiagbe (2017) | Yes | Yes | Yes | Yes | Yes | Yes | Yes | Yes | Yes | 9 |
| Antwi-Baffour (2023) | Yes | Unclear | Unclear | No | Yes | Yes | Yes | Yes | Unclear | 5 |
| Sarfo (2020) | Yes | No | Yes | Yes | Yes | Yes | Unclear | Yes | Yes | 7 |
| Alhassan (2022) | Yes | Unclear | Yes | Yes | Yes | Yes | Yes | Yes | Yes | 8 |
| Agyemang-Yeboah (2019) | Yes | Yes | Yes | Yes | Yes | Yes | Unclear | Yes | Yes | 8 |
| Adua (2017) | Yes | Unclear | Unclear | No | Yes | Yes | Yes | Yes | Unclear | 5 |
| Osei-Yeboah (2019) | Yes | Unclear | Yes | Yes | Yes | Yes | Unclear | Yes | Yes | 7 |
| Asamoah-Boakye (2017) | Yes | Yes | Unclear | No | Yes | Yes | Yes | Yes | Unclear | 6 |
| Brenyah (2013) | Yes | Unclear | Unclear | No | Yes | Yes | Yes | Yes | Unclear | 5 |
| Adu (2019) | Yes | Yes | Yes | No | Yes | Yes | Yes | Yes | Yes | 8 |
| Sarfo-Kantanka (2018) | Yes | No | Unclear | Yes | Yes | Yes | Yes | Yes | Unclear | 6 |
| Lokpo (2022) | Yes | No | Yes | No | Yes | Yes | Yes | Yes | Yes | 7 |
| Adjei (2024) | Yes | Unclear | Yes | Yes | Yes | Yes | Yes | Yes | Yes | 8 |
| Adu (2024) | Yes | No | Yes | Yes | Yes | Yes | Unclear | Yes | Yes | 7 |
| Mogre (2014) | Yes | Unclear | Unclear | No | Yes | Yes | Unclear | Yes | Yes | 5 |
| Swaray (2023) | Yes | Yes | Yes | Yes | Yes | Yes | Unclear | Yes | Yes | 8 |
| Adong (2024) | Yes | Yes | Yes | Yes | Yes | Yes | Unclear | Yes | Yes | 8 |
| Sisu (2021) | Unclear | No | Unclear | Yes | Yes | Yes | Unclear | Yes | Unclear | 4 |
| Yorke (2024) | Yes | Yes | Yes | Yes | Yes | Yes | Unclear | Yes | Yes | 8 |
| Djonor (2021) | Yes | No | Yes | Yes | Yes | Yes | Unclear | Yes | Yes | 7 |
| Addai-Mensah (2019) | Yes | Unclear | Yes | Yes | Yes | Yes | Yes | Yes | Yes | 8 |
| Apini (2018) | Yes | Yes | Unclear | No | Yes | Yes | Yes | Yes | Unclear | 6 |
