# Supplementary material for: Prevalence and sociodemographic determinants of suboptimal glycemic control in persons with diabetes in Ghana: A systematic review and meta-analysis
Source: PLoS One. 2025 Jul 18;20(7):e0327610. doi: 10.1371/journal.pone.0327610 (PMC12273950; doi:10.1371/journal.pone.0327610)
Supplement: S1 Fig — (DOCX) [file pone.0327610.s004.docx]

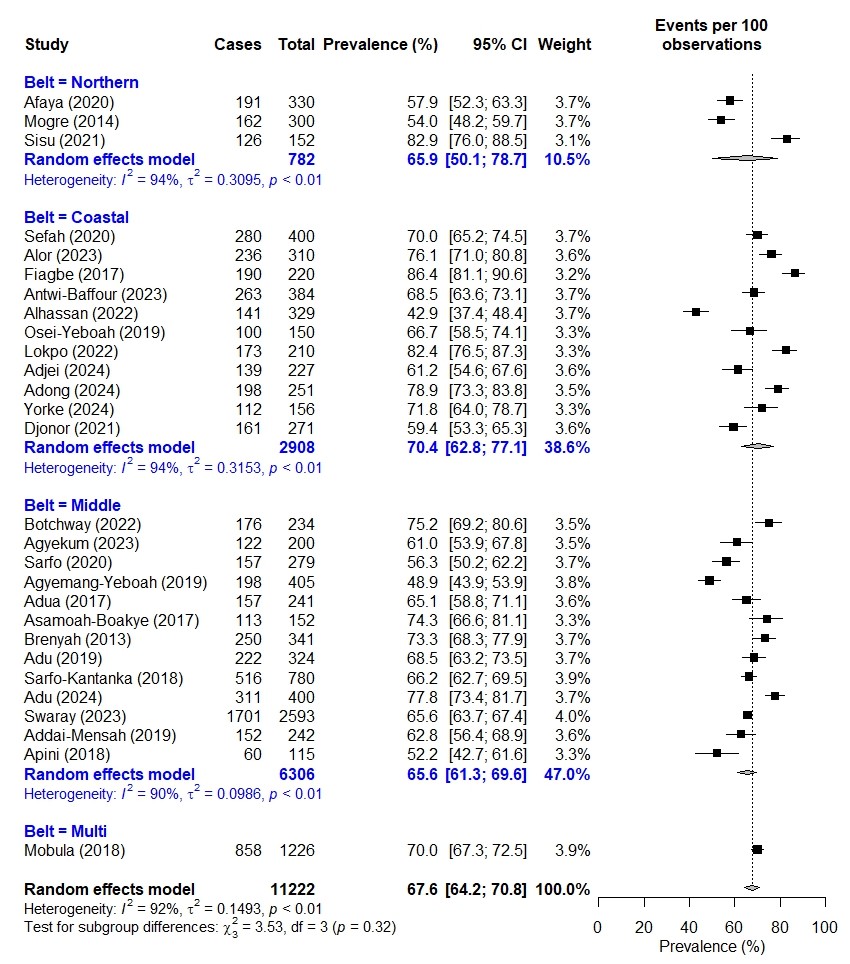


Forest plot for subgroup analysis based on geographic belts in Ghana


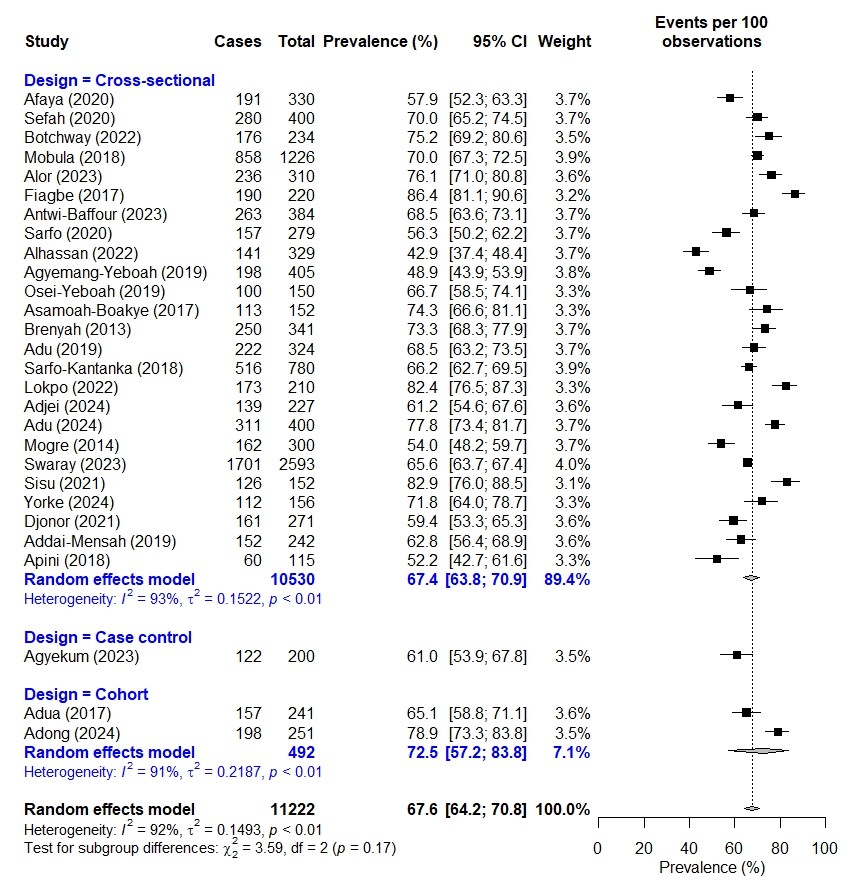
Forest plot for subgroup analysis based on study design


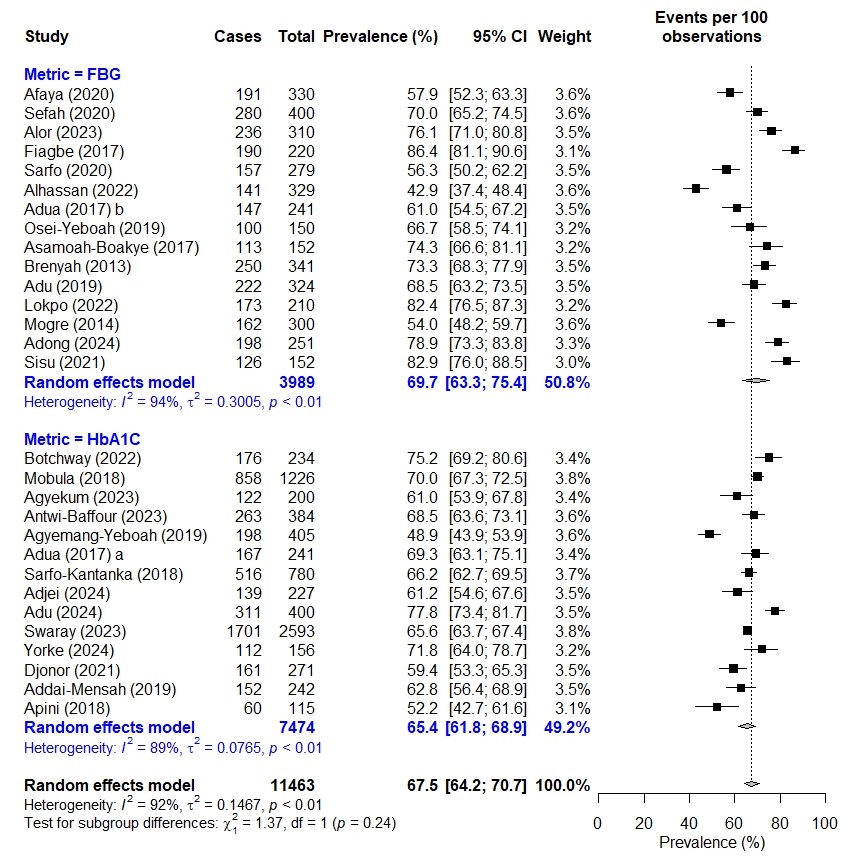


Forest plot for subgroup analysis based on glycemic metrics


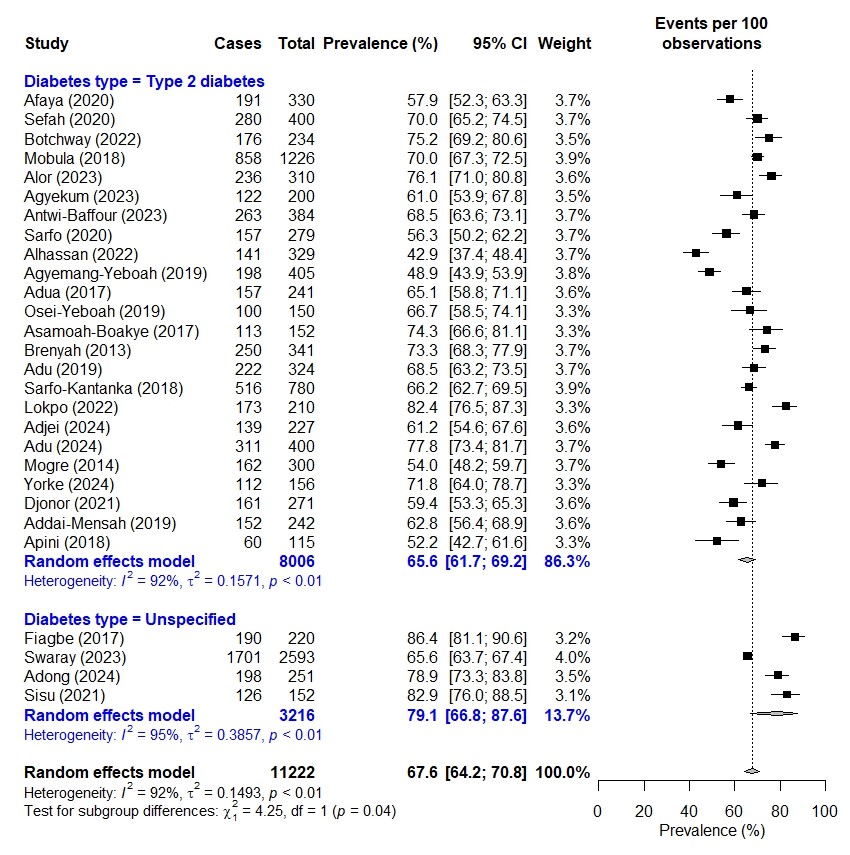
Forest plot for subgroup analysis based on diabetes type


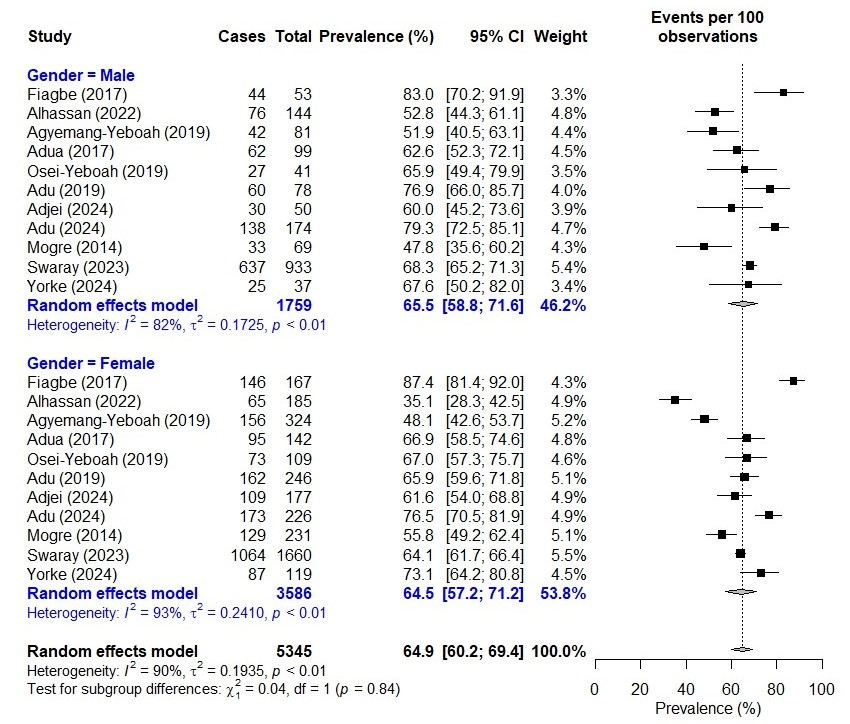


Forest plot for subgroup analysis based on gender
