# Supplementary figures and images for: Prevalence and sociodemographic determinants of suboptimal glycemic control in persons with diabetes in Ghana: A systematic review and meta-analysis
Source: PLoS One. 2025 Jul 18;20(7):e0327610. doi: 10.1371/journal.pone.0327610 (PMC12273950; doi:10.1371/journal.pone.0327610)

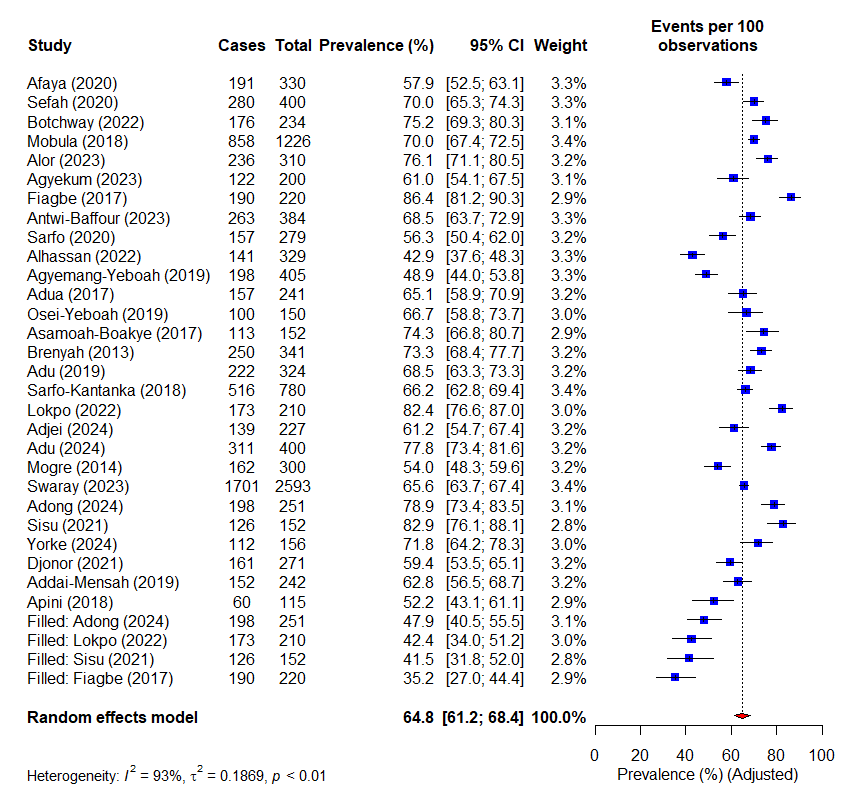


Forest plot for trim-and-fill analysis

Supplement: S2 Fig — (DOCX) [file pone.0327610.s005.docx]
